# Experimentally induced awe does not affect implicit and explicit time perception

**DOI:** 10.3758/s13414-019-01924-z

**Published:** 2019-11-25

**Authors:** Michiel van Elk, Mark Rotteveel

**Affiliations:** Department of Psychology, University of Amsterdam, Amsterdam, The Netherlands

**Keywords:** Awe, Emotion, Time Perception, Tactile Bisection Task, Subjective Feelings

## Abstract

The effects of emotion on time perception are elusive: depending on the intensity, valence and arousal of the situation, implicit and explicit time perception seems to slow down or speed up. Awe is a strong and powerful positive emotion that is typically elicited in response to vast stimuli and therefore inducing awe may be optimally suited for studying the relationship between emotion and time perception. In two studies we investigated whether the experience of awe would result in an expanded perception of time. Participants watched awe-eliciting, positive and neutral videos and simultaneously conducted a temporal bisection task, in which they classified vibrotactile stimuli as short or long. As expected awe videos elicited stronger feelings of awe than positive and control videos, while they were matched with positive videos in terms of subjective valence and arousal. However across both studies we did not find consistent effects of awe on implicit and retrospective time perception. Only in the first study, stronger subjective feelings of awe were associated with an increased dilation of time perception. The current findings indicate that lab-induced awe does not affect implicit and explicit time perception and we suggest that more ecologically valid ways to induce awe may be required in future studies.

**Electronic supplementary material:**

The online version of this article (10.3758/s13414-019-01924-z) contains supplementary material, which is available to authorized users.

## Introduction

The subjective experience of time is elusive. Time flies when watching an engaging movie or a spectacular concert. But time seems to slow down or even stand still, when walking in a beautiful natural environment or while practicing mindfulness meditation. What could account for these apparently contradicting experiences? In this paper we present the results of two studies on the effects of awe on time perception to shed light on this intriguing question. Building on theories of subjective time perception (Grondin, 2010), extending the established effects of emotion on subjective time perception (Droit-Volet & Meck, 2007) and based on the subjective experience that during awe (and other self-transcendent experiences) time seems to stand still, we hypothesized that especially the experience of awe should result in a clear overestimation of temporal intervals.

Awe can be triggered by a wide range of different circumstances and stimuli, such as the birth of a child, the experience of a beautiful landscape, reflection on the infinity of the universe or reading about great scientific achievements (Dworkin, 2013). What these examples have in common is the experience of perceived vastness, i.e., being confronted with something that is bigger than oneself or exceeds one’s expectations, resulting in an accompanying need for accommodation to revise one’s current mental structures (Keltner & Haidt, 2003). For instance, coming from the Netherlands where everything is flat and low, being confronted for the first time with natural beauty (e.g., while visiting natural parks in the US or Scandinavia) results in feeling small and insignificant and in a revision of one’s mental schemes (‘I didn’t know that nature could be that grandiose.’). Indeed it has been found that experimental manipulations of awe can foster the awareness of one’s knowledge gaps and a motivated interest in science (McPhetres, 2019). The effects of awe on scientific explanations also seem to depend on one’s religious worldviews (Valdesolo, Park, & Gottlieb, 2016). Thus, awe seems to be an epistemic emotion by fostering a search for knowledge and understanding (Valdesolo, Shtulman, & Baron, 2017).

Recent studies have indicated that awe can have beneficial consequences, such as fostering prosociality, well-being and forgiveness for instance (Piff, Dietze, Feinberg, Stancato, & Keltner, 2015; Rudd, Vohs, & Aaker, 2012; Zhang, Howell, & Iyer, 2014). Other studies have shown that awe makes people literally feel smaller and changes the perception of one’s body (Joye & Dewitte, 2016; Piff et al., 2015; van Elk, Karinen, Specker, Stamkou, & Baas, 2016). A previous study has provided preliminary evidence for the notion that awe directly affects time perception (Rudd et al., 2012). In Study 1 all participants were primed with the concept that time is limited (i.e., by using a scrambled sentence task), and following an awe-inducing manipulation they indicated their agreement with four items related to time availability (e.g., ‘I have lots of time in which I can get things done’).^1^ It was found that participants in the awe-condition scored higher on the time-availability condition than participants in the control condition. In a second study it was found that an awe-manipulation resulted in feeling less impatient and this effect in turn was related to a higher willingness to voluntarily give time. Finally, in a third study it was further confirmed that awe affects subjective perceptions of time availability and this effect was in turn related to life satisfaction.

A limitation of these studies however is that they focus selectively on prospective time perception (Grondin, 2010), i.e., the participants knew in advance that they were required to judge the perceived duration of time intervals. They alsoi relied entirely on self-report measures, thereby making the manipulations prone to socially desirable responding (van de Mortel, 2008). To remedy this problem, in this study we used implicit behavioral measures of time perception, while participants were actually feeling awe.

In the scientific literature, a lot of research has focused on the effects of emotions on both prospective and retrospective time perception, by using a variety of different behavioral measures (Droit-Volet & Meck, 2007) and this research has identified a number of factors that affect the perceived duration of stimuli. Most research in this field builds on a so-called internal clock model of time perception, according to which an ‘internal clock’ generates pulses that are accumulated until a comparator establishes that a certain period of time has passed (Droit-Volet & Meck, 2007). Although this model has been criticized for its lack of neural plausibility (M. Wittmann, 2013), it provides a useful framework to account for a wide range of different findings. The model also allows generating testable predictions regarding the different factors affecting time perception, and we will shortly review the available evidence for these hypotheses below.

First, several studies have shown that attention has an independent effect on time perception: when attention is diverted away from temporal processing (e.g., by focusing one’s attention on an engaging task or interesting stimuli) this results in an underestimation of the time that has passed, as less internal pulses are registered (Brown, 1997; Macar, Grondin, & Casini, 1994). Similarly: when participants listened to engaging musical stimuli, these were judged to be shorter in duration than control stimuli that were more boring (Droit-Volet, Bigand, Ramos, & Bueno, 2010). Thus, directing attention away from temporal processing when one is fully engaged or absorbed in an experience may result in an underestimation of the time that has passed (i.e., reflecting the well-known feeling that time flies when you are doing something you are really engaged in). Indeed, it has been shown that feelings of awe compared to the experience of interest and curiosity results in a broadening rather than a narrowing of one’s attentional scope – although the sample size in the critical study was small (Sung & Yih, 2016).

Second, many studies have shown that time perception is also affected by arousal, valence and stimulus complexity: more arousing stimuli, highly positive or negative stimuli, and complex stimuli result in an over-estimation of the time that has passed. An often-used paradigm to study time perception in relation to emotional stimuli and arousal is the temporal bisection task (Droit-Volet & Meck, 2007). In this task participants are first trained to distinguish between a short and a long stimulus presentation time and next they are required to classify stimuli of varying durations as being more similar to the short or the long stimulus duration. Using this task it has been found that angry faces were perceived as longer in duration than neutral faces, as reflected in a leftward shift in the bisection function (Droit-Volet, Brunot, & Niedenthal, 2004). Similarly, both positive and negative emotional stimuli were judged as being longer in duration than neutral stimuli (Noulhiane, Mella, Samson, Ragot, & Pouthas, 2007) and this effect has been related to an increase in arousal accompanying the processing of emotional stimuli (Mella, Conty, & Pouthas, 2011). Threat also affects temporal perception, as stressed rats showed a horizontal shift to the left in the bisection functions (Meck, 1983) and humans perceived moving towards dangerous cliff as longer than moving away from it (Langer, Wapner, & Werner, 1961). Watching threatening movies, but not neutral or sad movies resulted in a shift in the temporal bisection task compared to performance before watching the movie (Droit-Volet, Fayolle, & Gil, 2011). Similarly, direct manipulations of physiological arousal (e.g., as induced through intense visual stimulation or drugs) also resulted in an overestimation of time, possibly by speeding up the internal clock (Droit-Volet & Wearden, 2002; Wearden & Pentonvoak, 1995). More complex stimuli also result in judgments of longer duration (Schiffman & Bobko, 1974) and this has been interpreted as evidence that the brain estimates time intervals based on the number of events that have occurred (Brown, 1995).

Third, several studies have shown that the effects of emotion on time perception depend on individual differences in emotional experience. It has been found for instance that the temporal bisection bias in relation to threatening stimuli is related to self-reported negative emotions (Tipples, 2008) and that high-anxious individuals primarily display a temporal bias effect (Bar-Haim, Kerem, Lamy, & Zakay, 2010). Individual differences in approach-motivation have also been related to an altered perception of the duration of emotional stimuli (Gable & Poole, 2012). Thus, individual differences in the emotional intensity of an experience may moderate the effects of emotion on time perception.

Based on these findings different hypotheses can be formulated regarding the effects of awe on time perception. Awe is a captivating experience that fully captures one’s attentional resources, resulting in an absorptive state of mind (van Elk et al., 2016). Accordingly, it could well be that awe directs attention away from time perception, resulting in less pulses being registered and accordingly an underestimation of the time that has passed. At the same time, awe is a *highly arousing* and *positive emotion* (although it can also be a threatening experience; cf., Shiota, Keltner, & Mossman, 2007) that is elicited by *complex stimuli* (e.g., excerpts from BBC nature documentaries, showing rapid changes in natural sceneries). Studies using physiological measures have shown no acute effects of awe stimuli on physiological measures of arousal, such as respiration or skin conductance responses (Oveis et al., 2009; van Elk et al., in prep.), although more immersive awe experiences through a Virtual Reality manipulation were indeed associated with an increase in skin conductance (Chirico et al., 2017). Therefore it can be expected that the experience of awe results in an overestimation of time, reflected in a leftward shift in the temporal bisection function. Furthermore, given the relationship between subjective experience and time-perception it could well be that individual differences in experienced awe could moderate the effects of awe on time perception.

Thus, based on the existing theoretical notions different predictions could be formulated regarding the effects of awe on time perception, which were tested in two studies. We used different short videos to elicit awe. To control for the potential confound that potential effects of awe were merely related to valence and arousal, we included positive and neutral stimuli as a control condition (i.e., movies of funny animals and cars etc.) and in addition we asked participants following each video to provide a valence and arousal rating. Based on a pre-test the positive stimuli were matched in terms of valence and arousal to the awe videos, while they were rated as less awe-inducing than the awe-videos. The details of the pre-test and the selection of the stimuli can be found in a previously published fMRI study, using the same experimental stimuli and design (van Elk, Gomez, van der Zwaag, van Schie, & Sauter, 2019).

Because we were primarily interested in studying the online effects of induced awe on temporal judgments (i.e., while participants were actually engaged in watching the awe-eliciting movies), we used a bisection task while participants were watching the different videos. Specifically we used a tactile bisection task (Jia, Shi, Zang, & Muller, 2015; Shi, Jia, & Muller, 2012), in which participants categorized vibrotactile stimuli as being of short or long duration. The advantage of this task is that it does not interfere with the other sensory modalities (i.e., vision and sound) that are used to elicit awe to prevent any interference. Both studies shared a similar experimental design, by measuring implicit time perception by using the tactile bisection task. In the second study we additionally used an explicit measure of time perception, by using a retrospective time estimation task.

## Study 1

### Methods

#### Participants

In total 18 participants participated in this first exploratory experiment (12 females, mean age = 21.4, *SD* = 1.65 years), all students at the University of Amsterdam, the Netherlands. As there were no previous studies on this topic, there was no a priori information regarding the expected effect size to conduct a power analysis. This problem was addressed in Study 2 however. All participants gave written informed consent before participation and received 5 Euros or course credits for participation. The experiment was conducted in accordance with the guidelines from the Declaration of Helsinki.

#### Stimuli

As stimuli we used 30-second video clips depicting different scenes and objects. In the first pilot study we used a total of 24 different videos: 8 videos to elicit awe, 8 positive videos and 8 control videos and each video was presented once. The videos were pre-tested in a separate study in which 17 North American participants (8 females; mean age = 39.8 years) recruited through Amazon’s Mechanical Turk, rated a larger selection of videos for arousal, valence, awe, familiarity and the feeling of ‘chills’ on a visual analog scale ranging from 1 (‘not at all’) to 100 (‘very much). Based on the pre-test we selected 8 videos per category such that awe videos and positive videos were matched for valence, arousal and familiarity, while they differed on feelings of awe and chills (see Table 1). The videos that we selected were the same as those used for an fMRI study in which we aimed to identify the neural correlates of the awe experience (van Elk, Arciniegas Gomez, van der Zwaag, van Schie, & Sauter, 2019), in which further details of the pre-test are provided. As expected, positive videos were rated as more arousing (i.e., participants indicated that they felt less calm or relaxed) and more positive than control videos.

**Table 1:** Ratings from the pre-test for Awe, Positive and Control videos that were used in the pilot experiment

	Arousal	Valence	Familiarity	Awe	Chills
Awe Videos	43.9 (8.7)	84.9 (3.1)	2.1 (1.3)	73.5 (4.4)	40.4 (15.5)
Positive Videos	45.9 (2.1)	84.2 (4.0)	2.0 (1.7)	35.5 (8.1)	11.6 (3.1)
Control Videos	24.6 (3.5)	70.4 (7.6)	1.6 (1.5)	25.4 (7.8)	9.1 (3.4)

Standard errors are between brackets.

#### Experimental Design and Procedure

**Fig. 1:**
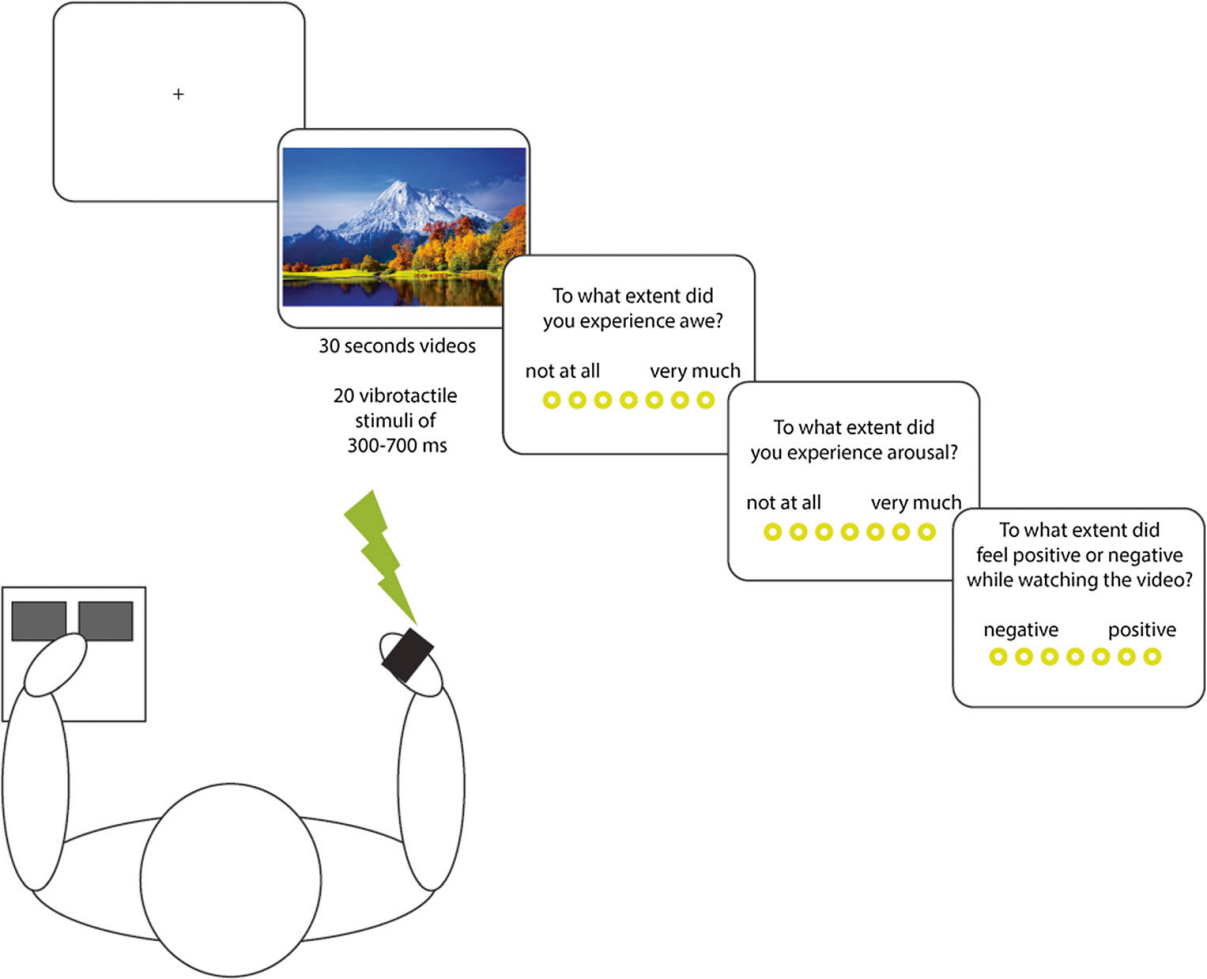
Overview of experimental setup and procedure. Participants were presented with 30-seconds videos during which vibrotactile stimuli of varying durations were presented. During the videos participants were required to classify the tactile stimuli as short or long by pressing one of two buttons. At the end of each video participants were asked to rate to what extent the video elicited feelings of awe, positive (vs. negative) feelings and arousal.

The experimental flow and procedure is presented in Figure 1. Participants were seated behind a table on which a video screen (resolution: 1920 x 1280 pixels; refresh rate = 60 Hz) and a keyboard were placed. A custom-made vibrotactile stimulator was applied to the participant’s right index finger to deliver short sequences of tactile stimuli during the presentation of the videos. Vibrotactile stimuli started 5000 milliseconds following the onset of the video. A sequence of 20 vibrotactile stimuli was presented during each video with a variable inter-stimulus interval of 500-1000 ms. The vibrotactile stimuli were presented with 10 different durations: 300, 350, 400, 450, 500, 550, 600, 650, 700 or 750 ms and each duration was presented twice during each video. Participants were instructed that during each video they would receive non-painful vibrotactile stimulation and that they had to respond to these stimuli by indicating with their left hand whether the vibrotactile stimulus was short or long. Before the start of the experiment participants were instructed about the experimental procedure (see Appendix for complete task instructions). They were instructed that they were about to watch 24 videos and that during each video they had to categorize tactile stimuli as being short or long. Following each video, participants were required to rate to what extent the video elicited feelings of awe, arousal and positive (vs. negative) valence on a 7-point scale.

At the start of the practice blocks participants were presented with reference tactile stimuli, consisting of two vibrotactile stimuli of a short duration (300 ms) and of a long duration (750 ms). After the instructions were clear, participants started with two practice videos that were not included in the main analysis and that were not presented in the main experiment. If everything was clear, the tactile reference stimuli were presented again as a reminder and next the experiment started. The videos were presented in two separate blocks - separated by a short break - and during each block in total 12 movies were presented, thus yielding a total of 24 different movies (8 for each emotion condition). After every four videos the tactile reference stimuli were repeated to remind the participants of the duration of a short and a long stimulus. The experiment was programmed using Presentation software (Neurobehavioral systems, Albany, CA, USA).

After the experiment was finished participants completed an online questionnaire, including demographic information, the Tellegen absorption scale (Tellegen & Atkinson, 1974) consisting of 34 items and the Dispositional Positive Emotion Scale (DPES; Shiota, Keltner, & John, 2006), consisting of 38 items. These scales were included for exploratory purposes, but given the relatively low number of participants in this study to assess individual differences, these data will not be analyzed or reported.

#### Data Analysis

The ratings for awe, valence and arousal in response to the different videos were transformed to a scale from 0 to 1 (0 = not at all / negative; 7 = very much / positive) and were analyzed using a repeated measures ANOVA with Video (awe, positive, neutral) as within-subjects factors (for similar analysis, see: Droit-Volet et al., 2010). The proportion of tactile stimuli classified as long (compared to short) was analyzed using a repeated measures ANOVA with the within-subjects factor Video (Awe, Positive, Neutral) and Duration (300, 350, 400, 450, 500, 550, 600, 650, 700 or 750 ms).

### Results

#### Video Ratings

**Fig. 2:**
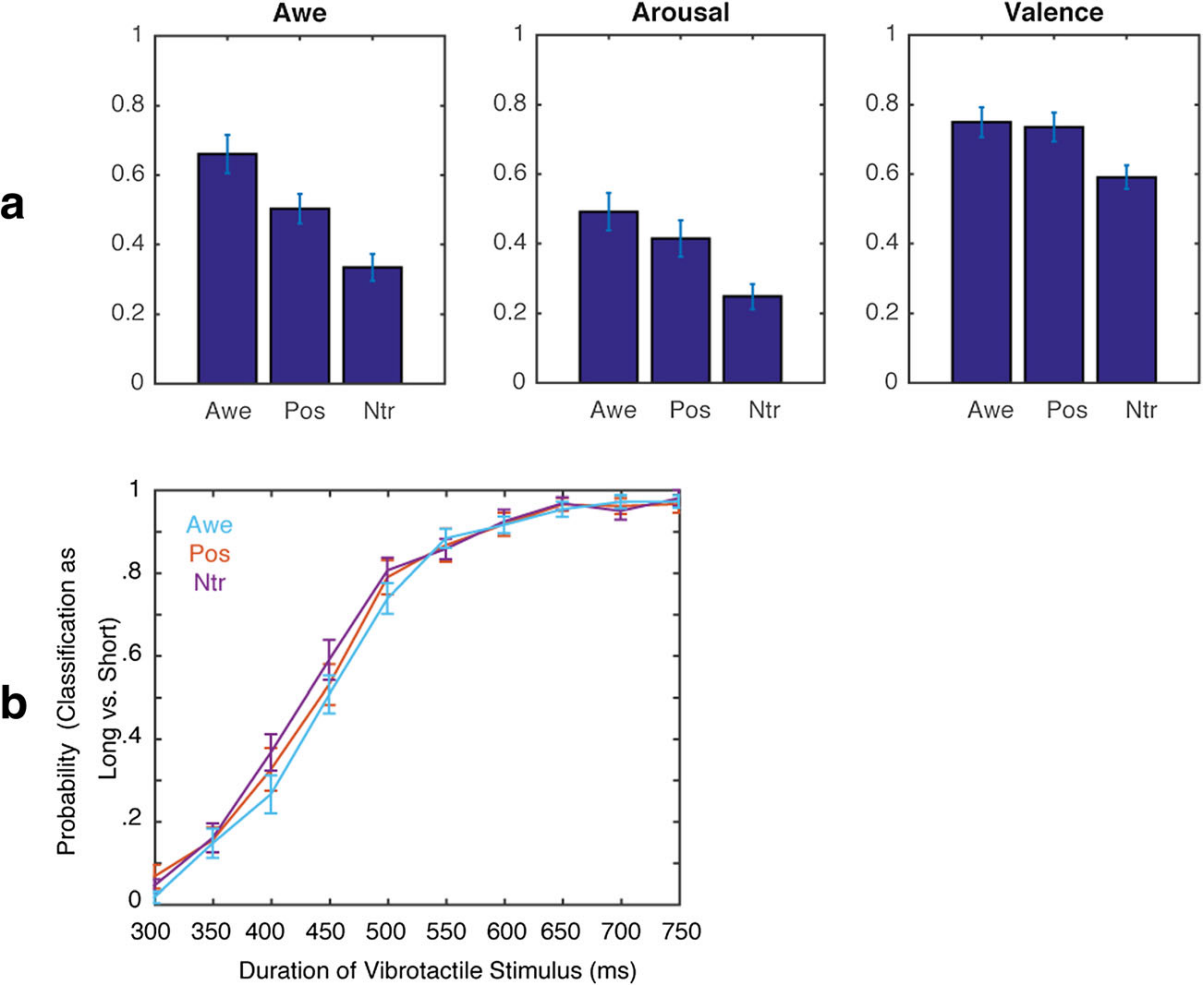
Results of Study 1. (A) Subjective ratings for awe (left graph), arousal (middle graph) and valence (right graph) for the different videos (Awe = awe videos; Pos = positive videos; Ntr = control videos). (B) Probability of classifying vibrotactile stimuli as long (compared to short) as a function of the duration of the vibrotatile stimulus for the different videos (Blue = Awe; Red = Positive; Purple = Neutral).

The ratings of the videos are represented in Figure 2A. For the *awe ratings* a main effect of Video, *F*(2, 34) = 27.40, *p* < .001, η^2^ = .62, reflected that participants experienced more awe while watching the awe videos (mean = .66; SE = .06) compared to the positive videos (mean = .50; SE = .04), *t*(17) = 3.45, *p* = .003, and compared to the control videos (mean = .33; SE = .03), *t*(17) = 7.00, *p* < .001. Positive videos were also rated as more awe-inducing than the control videos, *t*(17) = 4.36, *p* = .001.

For the *arousal ratings* a main effect of Video, *F*(2, 34) = 17.52, *p* < .001, η^2^ = .51, was found, reflecting that participants experienced more arousal while watching awe videos (mean = .49, SE = .05) compared to control videos (mean = .25, SE = .04), *t*(17) = 6.48, *p* < .001) and while watching positive videos (mean = .42, SE = .05) compared to control videos (mean = .25, *t*(17) = 3.66, *p* < .001). Awe videos were rated as somewhat higher on arousal (mean = .49) than positive videos (mean = .42) although the difference was not significant, *t*(17) = 1.81, *p* = .09.

For the *valence ratings* a main effect of Video, *F*(2, 34) = 15.70, *p* < .001, η^2^ = .48, was found, reflecting that participants perceived awe videos as higher in valence (mean = .75; SE = .04) than control videos (mean = .59; SE = .03), *t*(17) = 5.50, p < .001, and positive videos were rated as higher in valence (mean = .74; SE = .04) than control videos (mean = .59; SE = .03), *t*(17) = 5.24, *p* < .001. Awe videos and positive videos did not differ on valence ratings, *t*(17) = .39, *p* = .71.

#### Tactile Judgments

The probability of classifying a vibrotactile stimulus as long (compared to short) for the different durations of tactile stimulation and the videos is presented in Figure 2B. As expected, a main effect of Duration, *F*(9, 153) = 298.3, *p* < .001, η^2^ = .95, reflected that with increased duration participants more often classified the tactile stimuli as longer compared to shorter. The effect of Video was not significant, *F*(2, 34) = 1.30, *p* = .29, η^2^ = .07 and – contrary to our expectations - also the interaction between Video and Duration was not significant, *F*(18, 306) = 1.10, *p* =.35, η^2^ = .06. This was substantiated by a Bayesian repeated measures analysis, indicating that the null model was more likely than the alternative model given the data; (i.e., a BF_10_ = .206 for the effect of Video compared to the null-model and a BF_10_ = .001 for the interaction effect of Video and Time compared to the null-model).

**Fig. 3:**
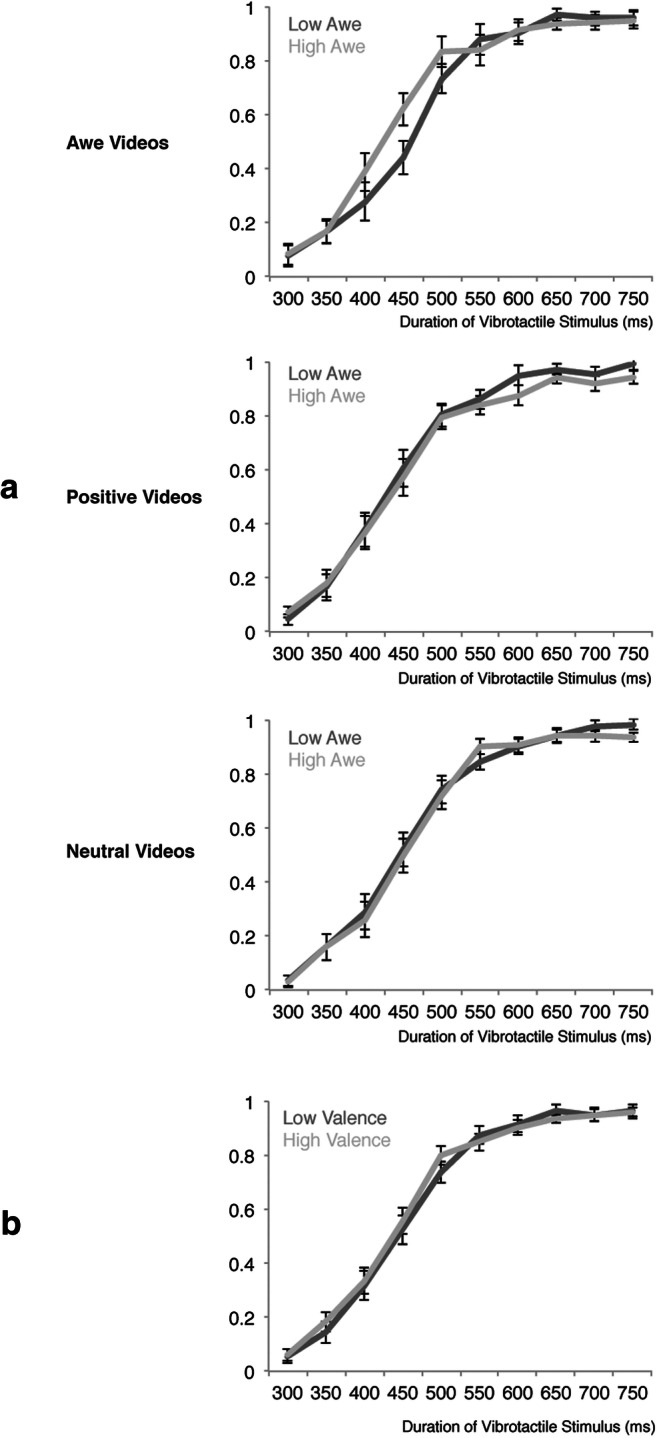
Probability of classifying vibrotactile stimuli as long (compared to short) as a function of the duration of the vibrotatile stimulus for the different videos (upper graph = awe; middle graph = positive; lower graph = control). Light lines represent participants scoring high on self-reported awe; dark lines represent participants scoring low on awe.

When including self-reported awe as covariate in the analysis, a significant three-way interaction was found between Video, Duration and Awe, *F*(18, 288) = 2.16, *p* = .005, η^2^ = .12. To visualize the directionality of this effect, a median-split was conducted on the subjective awe ratings, resulting in a group scoring overall low on awe (mean = .38, SD = .12) or high on awe (mean = .62, SD = .09; see Figure 3). When conducting separate post-hoc ANOVAs for each of the different videos, for the Awe videos a marginally significant interaction was found between Duration and Awe, *F*(9, 144) = 1.90, *p* = .056, η^2^ = .11, reflecting that participants who had a profound awe experience while watching the videos tended to classify stimuli of intermediate duration as longer compared to participants who experienced less awe (see Figure 3).

### Discussion

Our manipulation check indicated that the experimental manipulation was successful: participants experienced more awe when watching the awe-inducing videos compared to the positive and the control videos, while the awe videos did not differ from the positive videos in terms of valence and arousal. Although we found that awe videos were rated as somewhat higher on arousal than the positive videos, this difference was only marginally significant and could be related to a response bias as the different questions were always presented in the same order (i.e., awe > arousal > valence). However, contrary to our first hypothesis the different types of videos did not directly affect time perception. Only subjectively experienced awe interacted with time perception: participants who reported strong feelings of awe showed a leftward shift in the bisection function for awe videos compared to the other videos. This finding indicates that individual differences in subjective experiences may play an important moderating role in the effects of emotional stimuli on time perception (Tipples, 2008).

However, it could also be that awe does not have a particular strong effect on implicit time perception, but that it might still have an effect on the explicit and retrospective perception of time. Retrospective time perception is more sensitive to the subjective experience of the time that has passed and strongly depends on a reconstructive memory process (M. Wittmann et al., 2015). As such, retrospective time perception may be more sensitive to capture the subjective effects of awe on time perception than the online psychophysical task – and thereby it would allow us to compare both measures of time perception in relation to awe. To this end, we presented the same videos from Experiment 1 not for the entire duration, but we cut the videos at different time-points, resulting in a duration ranging from 20 – 30 seconds. The same experimental design was used as in Study 1, including three different types of Video and the tactile temporal bisection task as our dependent measure. In addition, in Study 2 we also asked participants following each video to estimate the duration of the movie, as a retrospective time estimation task. This allowed us to obtain an estimate of explicit time perception as well – next to implicit time perception, which was measured using the tactile temporal bisection task.

Based on these additional manipulations, the following predictions are made. First, similar to Study 1, we expected that subjectively experienced awe interacts with the effects of video on time perception and vibrotatile stimulus duration, such that participants reporting strong feelings of awe show the strongest effects on temporal judgments of intermediate duration. Second, we hypothesized that the effect of awe on retrospective time perception would be more pronounced than the effect on prospective time perception.

## Study 2

### Methods

#### Participants

For Study 2 we tested 29 participants, all students at the University of Amsterdam, the Netherlands. In the first study, the critical effect of interest comprised an interaction between two within-subjects factors (i.e., Video & Duration) and a Covariate (i.e., subjectively experienced awe). Conventional statistical packages do not provide an opportunity to conduct a power-analysis to determine the required sample size to establish the presence / absence of such an effect. Therefore, a custom-made procedure was used (i.e., implemented in the R package) involving a bootstrap power analysis for a two-way within ANOVA with a covariate. Shortly, this procedure involved a bootstrapping procedure using the observed data from Study 1, in which the observed power was calculated based on different sample sizes (i.e., 10, 15, 18, 20, 30 or 50 participants; i.e., individual data was selected with replacement). The power was calculated by running 1000 simulations per selected sample size to determine the proportion of studies that yielded a significant three-way interaction. At a sample size of N = 18 the observed power was .9, at N = 20 the power was .98 and at N =30 the power approximated 1.0. This power analysis indicates that with at least 29 participants, Study 2 is sufficiently powered to detect a similar effect as observed in Study 1 (especially since we aim to test a similar group of participants). All participants will give written informed consent before participation and receive 10 Euros or course credits for participation. The experiment will be conducted in accordance with the guidelines from the Declaration of Helsinki.

#### Stimuli

The same 24 videos of 30-seconds duration were used as in Study 1. For the retrospective time estimation task it is important to have videos of slightly different durations (i.e., as participants are required to subjectively estimate the perceived duration, without relying too strongly on memory cues based on the preceding video). To manipulate the duration of the video we varied the display time of each video by stopping the video randomly between 20 and 30 seconds after the onset of the movie. As a consequence, the number of tactile stimuli presented during each video was also dynamically adjusted based on the duration of the video. Similar to Study 1, following each video participants will be asked to provide their ratings of awe, valence and arousal, which allows us to investigate whether ratings of awe were comparable to Study 1.

#### Experimental Design and Procedure

The experimental flow and procedure is similar to Study 1. Vibrotactile stimuli started 5000 milliseconds following the onset of the video and depending on the duration of the video, a sequence of 13 - 26 vibrotactile stimuli was presented during each video with a variable inter-stimulus interval of 500-1000 ms. The vibrotactile stimuli presented had 10 different durations: 300, 350, 400, 450, 500, 550, 600, 650, 700 or 750 ms which were presented equally often during each condition. Participants were required to respond to the tactile stimuli by indicating with their left hand whether the vibrotactile stimulus was short or long. Following each video, participants were required to rate to what extent the video elicited feelings of awe, arousal and positive (vs. negative) valence on a 7-point scale. Importantly, the order in which the questions are presented will be randomized from trial to trial to avoid response bias. In addition, participants will be required to indicate the perceived duration of the video as a measure of retrospective time perception.

After the instructions were clear, participants started with two practice videos that were not included in the main analysis. After every four videos the tactile reference stimuli were repeated to remind the participants of the duration of a short and a long stimulus. The experiment was programmed using Presentation software (Neurobehavioral systems, Albany, CA, USA). No individual difference measures were included.

#### Data analysis

First, we analyzed responses to the manipulation check questions. The ratings for awe, valence and arousal in response to the different videos were analyzed using a repeated measures ANOVA with Video (awe, positive, neutral) as within-subjects factor. The proportion of tactile stimuli classified as long (compared to short) will be analyzed using a repeated measures ANOVA with the within-subjects factors Video (Awe, Positive, Neutral) and Duration (300, 350, 400, 450, 500, 550, 600, 650, 700 or 750 ms). In a subsequent analysis we included perceived awe as covariate in the repeated measures ANOVA. The perceived duration of the videos in the retrospective time perception task was analyzed using Video (awe, positive, neutral) as within-subjects factor. We expected to replicate the results obtained in Study 1 and in addition that participants would rate awe videos as longer in duration than positive and control videos.

### Results

#### Video ratings

**Fig. 4:**
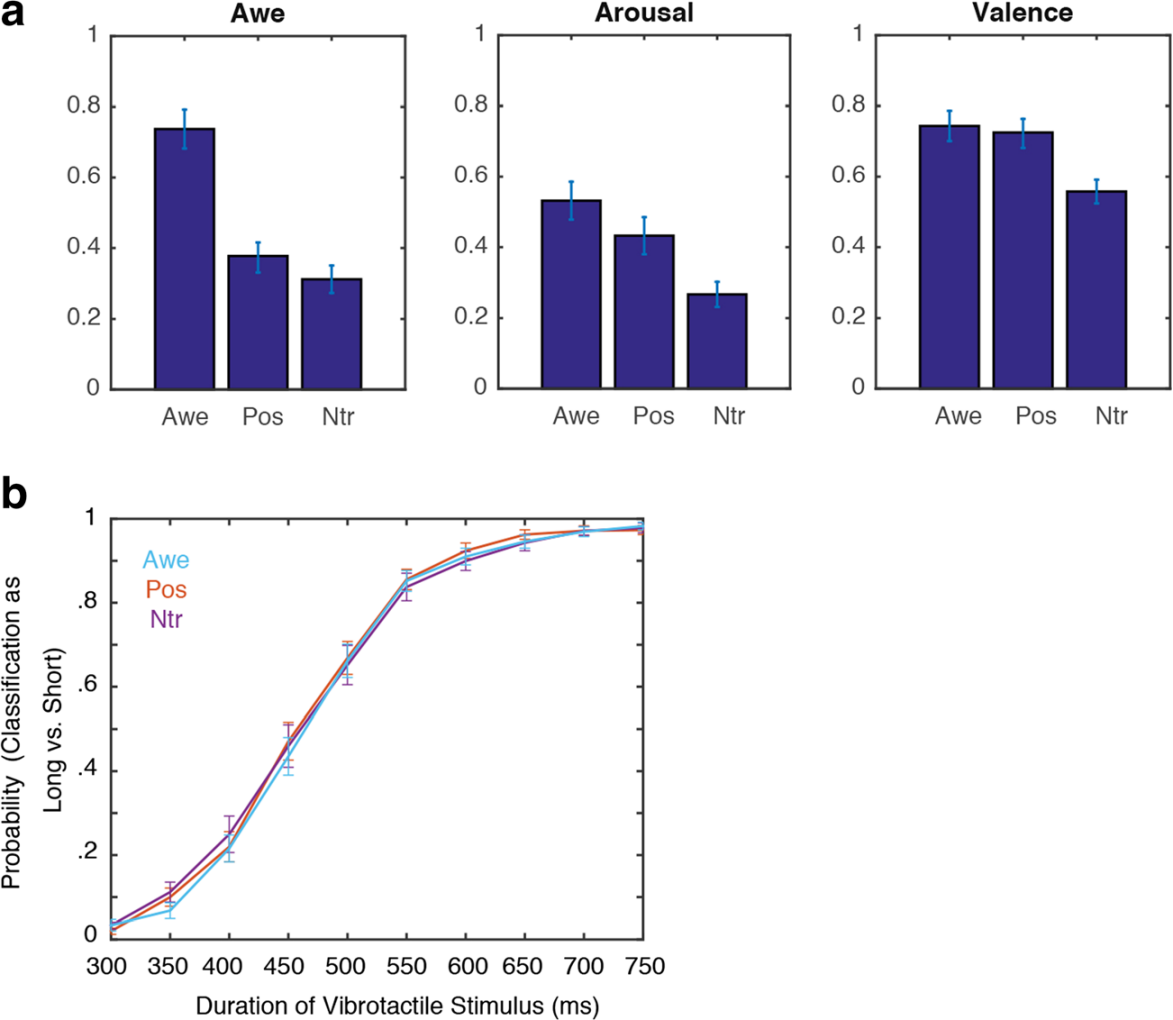
Results of Study 2. (A) Subjective ratings for awe (left graph), arousal (middle graph) and valence (right graph) for the different videos (Awe = awe videos; Pos = positive videos; Ntr = control videos). (B) Probability of classifying vibrotactile stimuli as long (compared to short) as a function of the duration of the vibrotatile stimulus for the different videos (Blue = Awe; Red = Positive; Purple = Neutral).

The ratings of the videos are represented in Figure 4. For the *awe ratings* a main effect of Video, *F*(2, 56) = 92.89, *p* < .001, η^2^ = .77, reflected that participants experienced more awe while watching the awe videos (mean = .74; SE = .03) compared to the positive videos (mean = .38; SE = .03), *t*(28) = 9.70, *p* < .001, and compared to the control videos (mean = .31; SE = .03), *t*(28) = 11.92, *p* < .001. Positive videos were also rated as more awe-inducing than the control videos, *t*(28) = 2.40, *p* = .023.

For the *arousal ratings* a main effect of Video, *F*(2, 56) = 27.96, *p* < .001, η^2^ = .50, was found, reflecting that participants experienced more arousal while watching awe videos (mean = .55, SE = .04) compared to control videos (mean = .27, SE = .04), *t*(28) = 6.66, *p* < .001) and while watching positive videos (mean = .43, SE = .05) compared to control videos (mean = .25, *t*(28) = 5.47, *p* < .001). Awe videos were rated as higher on arousal than positive videos although the difference was not significant, *t*(28) = 3.05, *p* = .005.

For the *valence ratings* a main effect of Video, *F*(2, 56) = 24.00, *p* < .001, η^2^ = .46, was found, reflecting that participants perceived awe videos as higher in valence (mean = .74; SE = .02) than control videos (mean = .56; SE = .02), *t*(28) = 5.66, p < .001, and positive videos were rated as higher in valence (mean = .73; SE = .03) than control videos (mean = .56; SE = .02), *t*(28) = 5.81, *p* < .001. Awe videos and positive videos did not differ on valence ratings, *t*(28) = .64, *p* = .53.

#### Tactile judgments

The probability of classifying a vibrotactile stimulus as long (compared to short) for the different durations of tactile stimulation and the videos is presented in Figure 4B. As expected, a main effect of Duration, *F*(9, 252) = 423.87, *p* < .001, η^2^ = .94, reflected that with increased duration participants more often classified the tactile stimuli as longer compared to shorter. The effect of Video was not significant, *F*(2, 56) = .44, *p* = .65, η^2^ = .02 and also the interaction between Video and Duration was not significant, *F*(18, 504) = .64, *p* =.87, η^2^ = .02. This was substantiated by a Bayesian repeated measures analysis, indicating that the null model was more likely than the alternative model given the data; (i.e., a BF_10_ = .02 for the effect of Video compared to the null-model and a BF_10_ = 5.23x10^^-6^ for the interaction effect of Video and Time compared to the null-model).

When including self-reported awe as a covariate in the analysis, in contrast to Study 1 the interaction between Video, Duration and Awe, *F*(18, 486) = 1.27, *p* = .202, η^2^ = .05, was not significant.

#### Retrospective Time Estimation

For the retrospective time estimation task, the effect of Video was not significant, *F*(2, 56) = 1.84, *p* = .17, *η*^*2*^ = .06, indicating that the type of video presented did not affect the estimated duration of the video (Awe videos: M = 19.78, SE = 1.30; Positive Videos: M = 20.93, SE = 1.39; Control videos: M = 20.01, SE = 1.41).

### Discussion

Our awe manipulation was successful: participants experienced more awe when watching awe videos compared to positive and neutral videos. Increased awe was also associated with feeling slightly more aroused. Still, similar to Study 1 we did not find an effect of awe on implicit time perception; nor did awe affect retrospective measures of time perception. This finding was contrary to our expectation that awe would result in an overestimation of time as measured using both implicit and explicit tasks. Also, we did not replicate the interaction between self-reported awe, video and time perception, which we observed in the first study. Thus, even when taking into account individual differences in the strength of the awe experience, time perception does not seem affected. Below we will discuss the implications and potential limitations of our findings.

## General Discussion

Across two studies we found that experimental manipulations of awe did not affect implicit and explicit time perception. Although in Study 1 we found an indication that subjective feelings of awe were associated with an over-estimation of temporal intervals, this effect was not replicated in a second study using a similar experimental design.

The absence of an effect of awe on time perception could be related to different concurrent processes contributing to the awe experience, related to attention, valence and arousal. The awe videos in the present study strongly captured visual attention as we observed in a previous study using the same stimuli (van Elk, Gomez, et al., 2019). The awe videos may have led to a reduced focus on time perception (i.e., less attention being paid to the tactile stimuli), resulting in less pulses being registered and an underestimation of the amount of time that has passed. On the other hand, the highly arousing and positive aspects of awe videos could contribute in an overestimation of the time that has passed and a leftward shift in the temporal bisection function. In the present studies both processes may have cancelled each other out, resulting in a complete absence of effects of video on time perception.

The absence of an effect of awe on time perception seems in apparent conflict with findings from a previous study, in which it was found that an awe manipulation induced the feeling in participants that they had more time available and felt less pressured (Rudd et al., 2012). However, in that study an explicit measure of *prospective* time estimation was used, while in our study we relied on an implicit measure of *retrospective* time perception. It could still well be that feelings of awe provide participants with the subjective sense that they have more time available. Previously reported effects may also be related to demand characteristics following reflection on the video and the questions that people were asked.

In this study we used a tactile version of the temporal bisection task as an implicit measure of the perceived duration of intervals, in which participants were required to classify tactile stimuli as being long or short. A major advantage of using a tactile version of the task is that it allows measuring implicit time perception, during the presentation of concurrent visual-auditory stimuli. The behavioral data showed that this task indeed resulted in a temporal bisection function, reflecting participants’ criterion for determining whether a presented vibrotactile stimulus was perceived to be short or long. The temporal bisection function was not modulated as a function of the type of video presented. It could be that the interval we used for our tactile stimuli was too short (i.e., 350-750 ms) to allow for sufficient variability in the perceived duration between conditions. Also contrary to our expectations, awe did not explicit time perception, as was measured in a retrospective time estimation task in Study 2. This could also be related to the relatively little variation in the duration of the different videos – as the time estimates showed that most participants were quite accurate in estimating the perceived duration of the videos. Furthermore, following the repeated presentation of stimuli, after the first trials participants were well aware that they were required to assess the perceived duration of each video, thereby potentially confounding retrospective with prospective time estimation processes.

The videos that we used in our study were relatively short and it might take more time to get fully absorbed in the stimuli in order to experience a dilation of time. Still in a previous fMRI study we found that using the same stimuli the awe-experience was accompanied by decreased focus on the self and an accompanying reduced activity of the default mode network (DMN), which has been implicated in self-referential processing and mind-wandering (van Elk, Gomez, et al., 2019). More powerful manipulations, e.g., by using immersive virtual reality (VR; see for instance: Chirico, Ferrise, Cordella, & Gaggioli, 2018; Chirico, Yaden, Riva, & Gaggioli, 2016; Reinerman-Jones, Sollins, Gallagher, & Janz, 2013) are likely to induce stronger feelings of awe, thereby enhancing the likelihood of detecting an eventual effect of awe on time perception. For instance, viewing earth from space in a virtual environment – as if floating through a space station – elicited strong feelings of awe; and immersive 3D videos of vast landscapes induced stronger feelings of awe than the 2D videos that are typically used in studies on awe (and in the present study as well). Additionally, the relationship between the awe-inducing stimuli and the time perception task could be made more salient, e.g., by asking participants to judge the duration of pictures that differ in their awe-inducing properties. Preliminary evidence indicates that people indeed tend to overestimate the duration of pictures representing natural compared to artificial objects (Berry et al., 2015).

In general, the experience of awe bears strong similarities to spiritual experiences (Van Cappellen & Saroglou, 2012; Van Cappellen, Saroglou, Iweins, Piovesana, & Fredrickson, 2013), which are also characterized by an altered perception of one’s body, the self and time (Piedmont, 1999). Awe could even be considered as one among a variety of self-transcendent experiences, including other states such as flow, mindfulness, peak experiences and mystical experiences (Chirico & Yaden, 2018; Yaden, Haidt, Hood, Vago, & Newberg, 2017). These experiences may vary along the unitary continuum, and can be characterized according to the degree of intensity of feelings of self-diminishment and connectedness (Yaden et al., 2017). Several strands of evidence, including neuroimaging studies on awe, meditation and psychedelics (van Elk, Gomez, et al., 2019), provide preliminary support for the notion of a common core associated with these self-transcendent experiences. It could well be that the sense of timelessness, which is characteristic of many mystical-like experiences (Hood Jr, 1975), is also a core feature of these experiences. Indeed it has been found for instance that extended practice with mindfulness meditation results in a leftward shift in the temporal bisection function, indicating a lengthening of perceived time (Droit-Volet, Fanget, & Dambrun, 2015; Kramer, Weger, & Sharma, 2013). Other studies have shown that both high and micro-doses of LSD, result in an overestimation of time, e.g., as measured by using a temporal reproduction task (Liechti, Dolder, & Schmid, 2017; Yanakieva et al., 2018). Thus, studying the changes in both implicit and explicit time perception in association with self-transcendent experiences seems to be a promising area of research.

## Conclusion

In sum, by using short videos (20 - 30 seconds) to induce awe in the lab, we found that feelings of awe were not consistently associated with an extended perception of time. Future studies on awe should try to disentangle the effects of attention and absorption in relation to the awe experience, use longer and more pronounced awe manipulations, and use dependent measures that allow to capture sufficient variability in time perception.

### Open practices statement

None of the data or materials for the experiments reported here is available, and none of the experiments was preregistered.

### Electronic supplementary material

ESM 1 (SAV 18 kb)

## Appendix

### Instructions presented to the participant before the start of the experiment

‘In this study you will be presented with a number of videos. During the video you will be presented with short vibrotactile stimuli. With the folloing buttons you are required to indicate whether the tactile stimulus was long or short:

Short: ←

Long: →

We will practice this task in a minute. Following each video you will be required to answer different questions. You can answer these questions by using the three arrow buttons on the keyboard (← = index finger; ↑ = middle finger; → = ring finger). You can respond by moving a circle on the screen to the left or to the right. You can confirm your answer by pressing the middle button. Please try not to think too long about your answer and respond as fast as possible.

After each video you will be presented with the following questions:

*Wonder:* To what extent did you experience feelings of awe or wonder while watching the video? Did you feel for instance: goosebumps, loss of sense of space and time, a strong connectedness with nature and humanity?

*Arousal:* To what extent did you feel bodily active or aroused while watching the video? Did you feel for instance: accelerated heartrate, moist hands, an active feeling?

*Valence:* To what extent did you experience positive feelings while watching the video? Did you feel for instance: happiness, cheerfulness; or did you feel: boredom, gloom, disgust?

We will now start with a short practice for the tactile task. During this task you will be presented only with short and long tactile stimuli. You can respond to the stimulus by pressing one of the following buttons:

Short: ←

Long: →

Please note: you should only respond to the stimulus when the buzzer has stopped.’

## Footnotes

1 We note that this study raises several questions regarding the methodology, e.g., ‘Why were all participants primed with the time-pressure concept and was there no control group included?’; ‘Which other ‘filler items’ were included to measure perceptions of time availability?’.
